# See Me, Hear Me, Know Me: Perspectives on Diet and Physical Activity Influences among Teens Living in Rural Texas Communities

**DOI:** 10.3390/nu15214695

**Published:** 2023-11-06

**Authors:** Debbe Thompson, Julie Miranda, Chishinga Callender, Jayna M. Dave, Godlove Appiah, Salma M. A. Musaad

**Affiliations:** USDA/ARS Children’s Nutrition Research Center, Department of Pediatrics, Baylor College of Medicine, 1100 Bates Street, Houston, TX 77030, USA; julie.miranda@bcm.edu (J.M.); chishinga.callender@bcm.edu (C.C.); jmdave@bcm.edu (J.M.D.); appiahgodlove67@gmail.com (G.A.); salma.musaad@bcm.edu (S.M.A.M.)

**Keywords:** rural, diet, physical activity, qualitative, barriers, facilitators

## Abstract

Teens in rural communities are at greater risk of obesity than teens in urban areas. Diet and physical activity influence obesity risk. Understanding their perspectives is an important step in intervention design. This qualitative investigation explored teen perspectives on how living in a rural community influenced their diet and physical activity choices. Forty parent–teen pairs were recruited. Data collection included surveys and telephone interviews. This paper reports teen perspectives identified in the first interview. Thematic analysis was used to code and analyze the data. Findings revealed that the primary factor driving teens’ diet and physical activity behaviors was the teens themselves. They clearly understood their role in the choices they made, although they acknowledged not always making the healthiest choice. This belief was driven by their motivation to engage in healthy behaviors, which was influenced by the perceived benefits derived from making healthy choices and from the synergistic relationship between diet and physical activity. Diet and physical activity, in turn, were influenced by the environment, particularly the home, social, and community environments. Family and friends were particularly influential, as well as resource availability. These findings can serve as a foundation for designing interventions tailored to this population.

## 1. Introduction

In the United States (US), 22.4% of 2–19-year-old youth have obesity [1] and 16.1% carry excess body weight [2], placing them at risk of becoming adults with obesity [3]. Once established, obesity is difficult to treat [4,5,6] or reverse [7,8], strengthening the case for primary prevention [9]. Disparities in prevalence of youth obesity exist, with certain groups of youth in the US at greater risk than others. For example, differences by birth sex have been observed, with 5–19 year old male youth more likely to be obese than their female counterparts [2]. However, an under-researched disparity is residential location, with rural residents often referred to as a “forgotten population” [10]. Children living in rural areas (i.e., areas not identified as an urban area or an urban cluster [11]) are more likely to be overweight or obese than children in urban areas [12,13]. A meta-analysis concluded that children living in rural areas have a 26% greater risk of being obese than children in urban areas, even after accounting for demographic characteristics [14], suggesting that the rural environment itself may be obesogenic [15].

Striking differences between urban and rural areas have been documented that may partially explain this disparity. Rural youth are more likely to live in poverty than those in urban households [16,17,18,19], which has been shown to be a key risk factor for obesity [20]. Furthermore, youth in rural areas are less likely to have access to physicians [17,21] and healthcare services [21,22]. They also face challenges in accessing healthy, affordable foods [23,24], being diagnosed with overweight or obesity [25], or receiving guidance on obesity-preventive behaviors such as healthy diet or physical activity [21,26]. Moreover, they are less likely to be aware of having excess body weight [27], which may influence their engagement in obesity-preventive behaviors [28] such as those associated with healthy diet and physical activity [29]. Limited access to food establishments with a variety of fresh, reasonably priced foods [22,30], and safe places to play such as parks and designated recreational areas [31], have also been reported. Alternatively, while the evidence is clear that youth living in rural areas face challenges that influence obesity risk, there are also protective factors that need to be considered. Rural residents often feel a strong sense of connection to family and community [12] and have rich cultural traditions [17] that may support behavior change.

Consuming an unhealthy diet increases risks of obesity, chronic diseases (e.g., cardiovascular disease, diabetes, several cancers [32]) and premature mortality [33]. Many teens do not meet physical activity guidelines [34], further increasing their risk [35]. In the US, cardiovascular disease, diabetes, and cancer are among the top ten causes of death [36]. Risk of developing cardiovascular disease, the leading cause of death in Western countries [37], is closely aligned with an unhealthy dietary pattern [38] and physical inactivity [35]. Thus, it is important to understand the food and physical environment from the perspective of the target audience to inform intervention development [39].

In summary, teens living in rural communities are at greater risk of obesity than those living in urban areas, and there is a lack of obesity prevention interventions tailored to this population. This is a concerning public health issue, as rural areas face unique challenges in addressing obesity among teens. To address this gap, comprehensive strategies that take these challenges into account from the perspective of the target audience are needed. This paper presents findings from a qualitative study investigating teens’ perspectives of how living in a rural community influences their diet and physical activity choices. This is a first step towards developing effective obesity prevention interventions for rural teens.

## 2. Methods

### 2.1. Sample

Teens (14–17-year-olds) and one parent were recruited for this study. This age range was selected because it represents middle adolescence, a key developmental stage characterized by physical and psychosocial changes and a drive for independence. These are key developmental tasks in preparation for the transition to adulthood [39,40]. Thus, middle adolescence is an important period in which to focus obesity prevention efforts. The Institutional Review Board at Baylor College of Medicine approved the research (H-46202).

### 2.2. Inclusionary Criteria

Eligibility criteria included: being 14–17 years old; having a parent/guardian willing to participate in the study; both parent and child having fluency in English or Spanish; primary residence in one of the targeted communities; access to the internet; and having an active email address or willingness to sign up for one. Exclusionary criteria included physical or mental conditions that would limit teen or parent study participation or unwillingness to have a telephone interview digitally recorded.

### 2.3. Recruitment

Recruitment procedures included identifying the rural counties in each of the four physical regions in Texas and the corresponding county zip codes. The concept of rural is defined in multiple ways, depending on the source. In a report issued by the Texas Legislative Council in June 2018, there were 46 different definitions of “rural” [41]. The Office of Management and Budget uses the terms metropolitan core (population ≥ 50,000), micropolitan core (population 10,000–49,999), and rural (non-metropolitan or micropolitan core). Any area not referred to as a metropolitan core is considered rural (i.e., this definition includes areas classified as both micropolitan core and rural) [42]. Using this definition as a guide, databases of rural counties and corresponding zip codes in the state of Texas were developed. When a family expressed interest in the study, their county was used to determine initial eligibility for the study. If their county included both metropolitan and rural areas, their zip code was then used to determine final eligibility.

To recruit families, we partnered with Prairie View A&M University (PVAMU) Cooperative Extension Program to distribute study flyers to families they served. To enhance program credibility, PVAMU’s logo was added to the flyer they distributed to families served by their organization. Additionally, study flyers were sent to organizations located in rural communities (i.e., libraries, doctors’ offices, YMCAs, waterparks/aquatic centers, Boys and Girls Clubs, school-based FFA programs). Brief study descriptions were also posted on the Baylor College of Medicine Facebook page, included in newsletters, and shared with other organizations that served families. A study coordinator described the study to interested families, answered questions, and screened for eligibility. Once written informed consent was received, the family was enrolled in the study. 

### 2.4. Data Collection

Data sources for the results reported here included online surveys and telephone interviews. Parents and teens completed a survey assessing demographic, home, and community characteristics. Following survey completion, teens also completed a telephone interview. Parents and teens were emailed a personalized link to complete the survey. Surveys were completed in REDCap, a secure, HIPAA-compliant data collection system. After completion of the survey, teens were contacted to schedule a telephone interview. To protect confidentiality, interviewers made the calls from a private room. Prior to the interview, teens were told that their name would not be mentioned during the interview in order to protect their confidentiality.

Prior to the telephone interview, the interviewer reviewed the survey responses to familiarize themselves with the teens’ responses, particularly those related to diet and physical activity behaviors. Interviews were guided by a script developed by the research team. Trained interviewers used probes and prompts to clarify, understand, and expand teens’ responses. Example of an interview question included, “In general, what places, if any, make it easy to make healthy [diet/physical activity] choices?” 

### 2.5. Data Analysis

Descriptive statistics were calculated for survey data. Parent and teen data were analyzed separately.

Interviews were professionally transcribed and reviewed for accuracy prior to analysis. The reviewed and corrected transcripts served as the data source for the qualitative analysis. Semi-structured thematic analysis, consisting of deductive and inductive codes, was used to code and analyze the data [43]. Initially, a codebook was created with deductive codes and definitions. As the transcripts were coded, inductive codes were identified, defined, and added to the codebook. Inductive codes helped ensure the participants’ perceptions and experiences were represented. 

Qualitative research is a rigorous research method that aims to understand a phenomenon or situation from the perspective of individuals who experience it. Thus, qualitative research is typically driven by a research question rather than a hypothesis [44]. The research question guiding this research was “How does living in a rural community influence diet and physical activity choices from the perspective of teens living in rural Texas communities?” Consistent with qualitative practices, coding was conducted to address this question.

Coding began with codebook and coder calibration. In this stage, the initial codebook was independently applied by two coders to one transcript. The coders met and discussed the codebook, definitions, and application of codes. Coding differences were discussed, and the codebook was updated as needed. Four additional transcripts were independently coded during this stage, and refinements were made to the codes and definitions as needed. At the end of this stage, coders were consistently applying the codebook to the transcripts. The remaining transcripts were then coded, with the coders routinely meeting to compare coding and reconcile any differences. If new codes were identified or definitions adjusted, all previously coded transcripts were re-reviewed to ensure the new or adjusted codes were consistently applied to all transcripts. At the completion of coding, transcripts were reviewed one final time to ensure codes were consistently applied. NVIVO 12 Plus (release date 2017) facilitated coding. After all transcripts were coded, codes were reviewed and grouped into categories, and categories were then grouped into themes and sub-themes. In the final step, themes and sub-themes were named. Qualitative analysis began in December 2022 and ended in July 2023. Themes and sub-themes were finalized in August 2023.

### 2.6. Rigor and Transparency

Rigor and transparency, core concepts in qualitative research, are similar to the concepts of reliability and validity in quantitative research [44]. They provide confidence in the findings and are the rationale for the rich descriptions of the target audience and the recruitment, data collection, and analytic stages typically seen in qualitative papers. In the research reported here, rigor and transparency were addressed by using trained interviewers, conducting enough interviews to reach data saturation, digitally recording the interviews, checking the recorder for audibility prior to the beginning of each interview, using verbatim transcriptions of the interviews, and comparing the transcripts to digital recordings to confirm accuracy prior to coding. Similar to past research conducted by this team [45,46,47,48], member checking (i.e., a qualitative procedure for obtaining participant validation to ensure accuracy and credibility) [49,50] was conducted throughout the interview to verify emerging patterns, trends, and observations. This was achieved by probing to clarify, understand, and expand responses. In the analytic phase, coding was conducted by two independent coders who routinely met to compare and resolve any coding differences. A codebook, containing codes and definitions, was maintained throughout coding to further ensure consistency in code interpretation and application.

## 3. Results

### 3.1. Recruitment

Recruitment began in September 2020 and closed in January 2023. Sixty-seven families expressed interest in the program. Of these, 11 were not eligible for reasons including the family not residing in the targeted county (*n* = 7), living out of state (*n* = 2), and the teen not being in the designated age range (*n* = 2). Of the 56 who were eligible, 40 enrolled. Reasons for not enrolling included families not responding to follow up calls (*n* = 16). The top recruitment sources were Cooperative Extension Agents (*n* = 15), word of mouth (*n* = 15), libraries (*n* = 9), and doctors’ offices (*n* = 8). Families were enrolled from 15 Texas counties, crossing the state of Texas from roughly its east/northeast to west/southwest borders. For example, border counties with families participating in the study included: Fannin (northeast; border shared with Oklahoma; median household income, USD 59,686; persons per household, 2.6; poverty rate, 14.3%); Shelby (east; border shared with Louisiana; median household income, USD 44,504; persons per household, 2.7; poverty rate, 19.6%); and Maverick (west; border shared with Mexico; median household income, USD 44,502; persons per household, 3.3; poverty rate, 20.5%) [51]. 

Further, examination of county-level data indicated that the 15 counties in which participating families lived were diverse. Population per square mile averaged 38.5 (range = 6.5–84.3 people per square mile); 86.6% of households owned a computer (range = 71.6–94.2%); and 77.2% had a home broadband subscription (range = 65.0–88.8%). On average, 25.6% of families reported speaking a language other than English at home (range = 8.2–58.2%). Average travel time to work was 26.8 min (range = 20.4–40.2 min), median household income was USD 50,375 (range = USD 25,000–USD 63,380), and 17.6% of homes reported including someone with a bachelor’s degree or higher (range = 12.7–29.1%) Total land mass averaged 900.4 miles (range = 544.5–1328.9) [51]. On average, 24.1% of 0–17-year-olds in these counties lived in poverty (range = 15.9–36.5%) [52]. The physician population in each county varied, with an average of 23.4 physicians per county (range = 3–103). Two counties reported over 100 physicians; excluding these two counties, the average physician population decreased to 11.3 per county (range = 3–35). Five counties reported having 3–5 physicians [53].

### 3.2. Participant Characteristics

Forty parent–teen dyads enrolled in the study. Parents were 30–49 years old (75.0%), married (82.5%), female (82.5%), White (90.0%), and non-Hispanic (57.5%) (Table 1). 

Parents reported that up to 2 adults (75.0%) and 2 children (75.0%) lived in the home, and at least 1 adult living in the home had a college education or higher (≥college education, 75.0%). Household income for most families was >USD 61,000 (70.0%), all families owned a vehicle (100.0%), and most spoke English at home (97.5%) (Table 2). For context, the average household income in the United States in 2021 was slightly less than USD 75,000 [54]. Additionally, most parents reported their family lived in a house (90.0%), while a few reported their family lived in a mobile home (5.0%), apartment (2.5%), or condominium/townhouse (2.5%), and 60.0% reported that their child received free lunches at school. While most parents reported that over the last 12 months their family had enough to eat and the kinds of foods they wanted (62.5%), others reported not always having enough to eat (5.0%), or that they had enough to eat, but not always the kinds of foods they wanted. Experiencing hunger one or more times over the last 12 months was reported by 10% of the parents. 

Most families drove ≤10 miles to food shop (67.5%) at a grocery store (95.0%) at least once a week (90.0%). Parents reported that families ate at least 3 meals together Monday through Friday (85.0%).

Teens were 14–15 years old (57.5%), female (60.0%), White (87.5%), and non-Hispanic (55.0%). Most played on 1 or more sports teams (75%) and reported being physically active for 60 or more minutes a day, at least 4 days a week (67.5%). Most described their current body weight as “about right” (52.5%), while some reported being slightly (30.0%) or very (2.5%) overweight. Alternatively, some reported being slightly (12.5%) or very (2.5%) underweight.

### 3.3. Qualitative Findings

**Figure 1:**
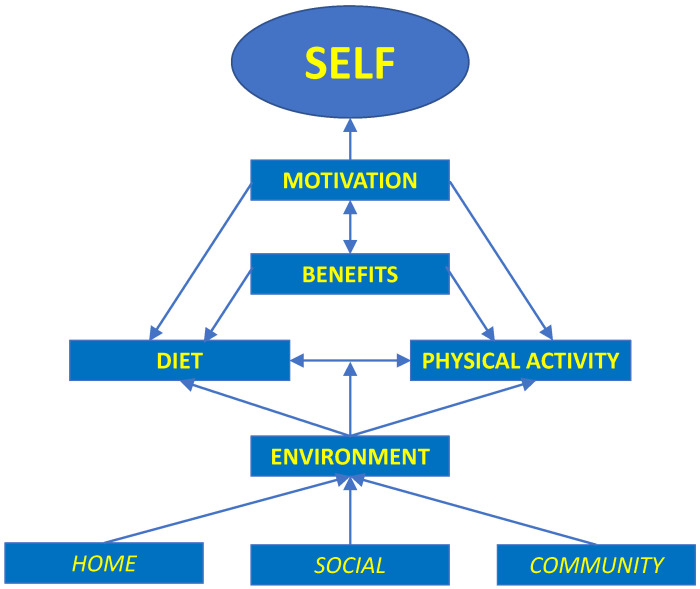
Thematic map of themes and sub-themes of factors that drive diet and physical activity behaviors of teens living in rural Texas communities.

A thematic map representing themes and sub-themes of factors that influence diet and physical activity choices of the teens participating in this study is presented in Figure 1. To aid in interpretation, themes are presented in bolded font and sub-themes are non bolded and italicized. The thematic network is described in more detail below, with a particular emphasis on diet, physical activity, and perceived benefits, as these themes emerged as important influences on motivation and teens’ overall sense of personal responsibility for diet and physical activity behaviors. Findings are supported by representative quotes. To maintain anonymity, quotes are identified by birth sex and age. 

Qualitative analysis revealed that the primary factor driving diet and physical activity choices of teens living in rural Texas communities was the teens themselves. They clearly understood their role in the choices they made, although they acknowledged not always making the healthiest choice. For example, one teen said: *…some days you just…don’t want to push yourself to go and do something that’ll be better for you. You know, you just want to be lazy and just lay around or whatever (14-year-old male).* They acknowledged they could be around people or places and in situations that made it challenging to make healthy choices, but clearly understood the final choice was up to them. This belief was driven by their motivation to engage in healthy behaviors, which was influenced by the perceived benefits associated with making healthy choices and the synergistic relationship between diet and physical activity choices.

The environment exerted a strong influence on diet and physical activity behaviors. Environmental influences for both diet and physical activity were grouped into three subthemes–home, social, and community environments. Each is discussed below, supported by representative quotes to provide context.

#### 3.3.1. Environmental Influences on Diet

Home environment. Teens viewed their mother as the primary influence on the home food environment. Mothers purchased and prepared foods, provided support and encouragement, and reminded the teen to make healthy choices. For example, a teen mentioned that (at their house they) *don’t have much junk food, so that is one, like, major thing because I would be eating lots of junk food, but there isn’t too much junk food. So that leads to good habits. I eat, like, better because there’s no junk food (17-year-old male).* Mothers not only played a role in food preparation, but also provided support by encouraging teens to eat healthily. As expressed by another teen: *She [mother] just really, like, I guess, emphasizes that eating healthy is important (15-year-old male)*. Alternatively, if the mother did not make healthy choices herself nor purchased or prepared healthy foods, it was more difficult for the teen to make healthy food choices at home. As one teen expressed: *My mom is a big one [*influence*]. I don’t think she does it intentionally, but she just–we don’t like to cook a lot, and she really doesn’t. So, we usually end up, not usually, but we eat out a lot. And when it is at home, it’s, like, quick stuff (14-year-old female).* In addition to the mother, other family members also influenced the home food environment by modeling healthy behaviors. For example, one teen mentioned: *My dad doesn’t eat any sugar for…like, the past 20 years. So since he makes like healthy eating choices…that’s, kind of, like, the food we have. So that’s the food I eat (17-year-old female).* Siblings were also influential, with one teen explaining how her older brother’s habit of eating fruit in the morning *influences…eating habits here at home (17-year-old female).*

Social environment. The teens’ social environment was influenced by friends, school, and coaches. In general, teens emphasized that it was easier to make healthy food choices when they surrounded themselves with people who also valued healthy eating. For example, a teen expressed: *And so it’s, kind of, like, also on fitting in with other people that choose to eat healthy. It makes—Like if your friend group does it, then you, kind of, want to do it too (14-year-old female).* Interestingly, friends could both facilitate and hinder healthy food choices. For example, one teen described how she tried a new food that had fruits and vegetables she would not usually eat because she saw *… everybody else trying it. So I was like, oh, well, I’ll give it a try. But it was pretty—It was really good, considering I didn’t like a lot of those fruits. Or fruits and vegetables (17-year-old female).* Alternatively, another teen mentioned that friends made it difficult to make healthy food choices because *they don’t work out or do sports, so it’s, like, it’s hard going to their houses because they usually don’t have a lot of healthy stuff there (14-year-old female).* The influence of schools was also discussed. Teens acknowledged that schools provided salads and fruit and have to follow guidelines that make the food healthy. However, they often did not like the food served and chose the snack line instead, which was less healthy: *The food that they [*school*] offer, like, in the actual food lines in our cafeteria, is not that great tasting. So I usually don’t eat that. And then they have a snack line, and it’s, like, chips and ice cream and cookies, none of it’s really healthy (14-year-old female).* Teens also acknowledged that they purchased unhealthy snacks at school because they were readily available, accessible, convenient, and tasted good.

Community environment. In general, teens perceived the community environment to be a barrier to making healthy food choices due to limited resources to support such choices. They mentioned that there were numerous places to purchase unhealthy foods, such as fast-food restaurants, truck stops, and sit-down restaurants, but there were only limited options for making healthier choices. Subway was often cited as a fast-food restaurant where they could opt for healthy selections: *The only healthy fast-food place there is, I would say is Subway. So we have McDonald’s, Dairy Queen. We have lots of fast food. So, you know, when I’m hungry and I really would rather prefer fries, you know, sometimes you’re craving fries. So yeah, I’d say my town. I don’t think there’s enough healthy places here. Only Subway (17-year-old male).* Although teens acknowledged the presence of grocery stores in their community, these establishments were not perceived as offering many healthy options: *Our grocery stores are smaller than you would find in a bigger city, so they’re going to have less of the newer vegetarian options (14-year-old female).* In addition, these stores were not viewed as a ready-source of meals or snacks: *As there’s not a whole lot of like grocery stores, like they’re pretty far away, like we have to rely on what’s around us and what’s around us is like the gas station and maybe like one deli, and the deli is kind of expensive. So like basically everybody, kind of, just goes to the gas station* (*15-year-old male*).

#### 3.3.2. Environmental Influences on Physical Activity

Home environment. Family was identified as the primary home environmental influence that made physical activity easy. Fathers and siblings were identified as equally influential, with mothers mentioned less often. Teens expressed that parents served as both role models for physical activity and sources of emotional support: *My parents are active; they like to run and workout…. always encouraging us to, you know, go outside, do some sort of dance. All of my family is in sports (17-year-old female).* Fathers primarily supported physical activity through co-participation and modeling. For example, one teen stated that he and his father *go to the gym together (15-year-old male),* while another said: *He [*father*] used to play sports a lot and I think sports is fun, so I wanted to play (14-year-old male).* Alternatively, family members, particularly siblings, could make it difficult to be physically active: *My older brother…always wants me to, like, play the X-Box with him a lot (14-year-old male).* Another said that their overall home environment influenced physical activity: *We spend a lot of time outside. I live on a large piece of land. So, like, pretty much all of my time at home when I’m not sleeping, it’s outside* (*14-year-old female*). In support of this, another teen mentioned: *We have a ranch and there’s cattle and horses. So, we’re always, like, moving around, staying active, you know. Fixing fences, stuff like that* (*15-year-old male*).

Social environment. The social environment for physical activity was comprised of friends and others with whom teens interacted, such as coaches and fellow athletes. Friends exerted a substantial influence on physical activity, particularly members of the teens’ general friend group, including best friends. Similar to the family, teens felt friends primarily encouraged physical activity through social support, particularly co-participation and modeling: *Me and my best friend, we’ll, like, go walking. We’ll walk or we’ll work out (17-year-old female).* However, friends could also make it difficult to be physically active, primarily because they preferred non-active behaviors: *My friend…we always plan to do something, and [then] we just lay in bed…and watch TikTok* (*16-year-old female*). An environment that was often mentioned as impeding physical activity was situations where being active was not feasible, e.g., vacation or travel; for example, as one teen said: *When I’m not in town…or at a friend’s house where there’s no gym* (*16-year-old male*). This seemed to be a result of the conflict caused by wanting to spend time with the person but also wanting to be physically active. Oftentimes, the teens felt they had to make a difficult choice between the two. Competing activities also influenced whether they were physically active. The teens seemed to have responsibilities or commitments that often conflicted with being physically active, such as work and a busy schedule: *Sometimes, I don’t have time to go work out (15-year-old female)*.

Community environment. Teens perceived that it was easy to be physically active in a rural community. The most often mentioned places that facilitated physical activity were their communities/neighborhoods and their schools. This appeared to be primarily driven by convenience, availability, and opportunity. As one teen said: *I have really easy access to a local park* (*17-year-old female*). Schools often made their facilities available to the community, as exemplified by one teen: *They [*the school*] have their facilities for working out and running, and the football field…has a track surrounding it … open to the public 24/7 (16-year-old male).* Another said: *My school is very athletic-centered. So just coming to school makes it super easy (15-year-old female*). In addition, a positive environment where others, including friends, family, and community at large, were engaged in physical activity served as an encouragement for teens to be physically active themselves. A positive environment also included events that encouraged physical activity, such as participating in a race to raise fundsfor a worthy cause. This suggests that teens living in a rural community perceived being physically active as a normative behavior—it is part of daily living. As one teen stated: *I would say just, like, a positive environment. Like being motivated by myself and seeing other people run. It just helps me get motivated more (16-year-old female).*

#### 3.3.3. Synergistic Relationship between Diet and Physical Activity

Teens in the study recognized a synergistic relationship between their diet and physical activity behaviors. For example, a teen who was a member of the school’s football team said being part of the team enabled him to have access to healthy food: *After workouts, they’re always giving us, like, these healthy fruits and, like, protein bars, protein shakes, stuff like that (17-year-old male).* Another teen emphasized the role of coaches in promoting healthy diet choices by offering reminders to eat healthily: *We’re one team, so make good eating choices so we can perform better (16-year-old male)*. Other teens discussed how coaches facilitated healthy dietary behaviors by offering encouragement: *So, the coaches at my school, they make it really easy ‘cause they motivate us to eat healthy and make sure we’re eating enough (17-year-old female)*. Finally, eating a healthy diet encouraged some teens to be physically active: *[*Working out*] makes you want to crave something a lot healthier in a diet (15-year-old male).* Alternatively, other teens understood the relationship between food and performance: *Like, if I’m at like a soccer game maybe….the day before, like, a soccer game, I … eat, like, some really, like, healthy foods… It helps fuel my body, but at the same time, like, I can enjoy the foods….(17-year-old male)*.

#### 3.3.4. Perceived Benefits

Similar benefits were observed for both diet and physical activity including improvements in overall health, appearance, and mental health. However, the order of importance of the benefits differed between the two behaviors. For diet, the most often-cited benefit was improved overall health, followed by appearance and mental health. Teens stated that foods consumed influenced both current and future health and gave them more energy. Additionally, they considered a healthy diet to positively influence their appearance, which teens implied meant *being in shape (16-year-old male).* Finally, they recognized mental health benefits associated with eating healthily, including that it helped them be *calm (17-year-old female)* and *less foggy (15-year-old male).*

When asked about the benefits of being physically active, nearly all teens highlighted the positive impact on mental health, such as stress management and attitude enhancement. For example, as one teen stated: *Whenever I’m stressing, exercising helps (17-year-old female),* while another said: *You feel good and you feel more productive (17-year-old male).* Overall health benefits were also mentioned, including being healthy and improved strength and muscle tone: *Your body like grows stronger (16-year-old-female)* and *…You’re always top energy and always doing stuff that helps your muscles grow and keeps you healthy and looking and feeling great (17-year-old female).* Appearance was also seen as an important benefit by some teens: *It’ll keep your body in shape or make you get into shape after a while (15-year-old male).*

## 4. Discussion

This work provides valuable insights into the perceptions of factors that influence diet and physical activity behaviors of teens living in rural Texas communities. The primary insight contributed by this research is the strong conviction teens who participated in the study expressed towards understanding their central responsibility regarding the choices they make. Furthermore, this study highlights the importance of mothers in influencing the home food environment; the influence of fathers, siblings, and friends on teen physical activity; and the role of availability in influencing both diet and physical activity behaviors.

Contrary to the literature, only a few parents reported being food insecure or hungry due to limited funds to purchase food. This is consistent with a recent report indicating that food insecurity was highest in principal cities located in large urban areas compared to rural areas [55]. The National Health and Nutrition Examination Survey between 2013–2016 found that elementary aged children living in urban areas were significantly more likely to live in households experiencing food insecurity compared to their rural counterparts [56]. This suggests that the rural context may offer certain advantages in terms of food security. More research in this area is warranted. 

Teens revealed that mothers and home availability were key factors that influenced the foods they ate. This finding aligns with other research that has identified the central role of mothers and home availability in shaping dietary behaviors [57,58,59,60,61]. Therefore, our finding is not surprising. What sets this study apart, however, is the conviction teens had regarding their central role in making healthy choices regardless of people, places, and situations that may make it more challenging.

An important finding regarding physical activity was the key roles played by fathers, siblings, and friends in shaping physical activity behaviors of teens. This finding is supported by previous research that highlighted the importance of fathers, particularly through co-participation, in promoting physical activity among children and adolescents [62,63]. It is important to note that, while family and friends can facilitate physical activity, they can also make it difficult when not active themselves. Others have reported similar findings [64,65,66,67]. This dual influence highlights the importance of social networks in shaping physical activity behaviors.

Another key finding was the relative ease of being physically active in rural environments compared to the difficulty accessing healthy foods outside the home environment. This supports findings by others that access to healthy, affordable foods may be more challenging in rural communities [22,23,24,30]. Therefore, interventions aimed at promoting healthy diet and physical activity behaviors may need a greater focus on overcoming barriers to eating a healthier diet or helping teens develop skills to make healthy dietary choices when faced with limited healthy options.

This research has important implications for research and practice. The findings highlight the importance of tailoring obesity prevention interventions to the unique needs and perspectives of teens in rural communities. Primarily, such interventions must emphasize personal choice and responsibility. Further, program leaders should clearly identify teen perceptions of the benefits associated with healthy choices, a key contributor to motivation, and strategically emphasize perceived benefits throughout the intervention. Finally, intervening at the environmental rather than the personal level may be important. For example, this could include identifying and promoting resource availability to support diet and physical activity, encouraging teens to identify individuals within the family and close friend group who can support them in making healthier diet and physical activity choices, and emphasizing how to make healthy choices in different environments (i.e., making healthy diet choices in a fast food restaurant; finding ways to be physically active when traveling). During intervention development, understanding the perspective of the audience of interest is an important first step in research designed to address health disparities such as those regarding obesity risk in rural communities. In participatory research, the end user is closely involved from the beginning, and their thoughts and perspectives are actively solicited to develop a deeper, more nuanced understanding [68]. Qualitative research provides a key framework for conducting participatory research in that it seeks to understand and give voice to participant perspectives [44], often leading to key insights and shared understandings of a particular topic [68]. Because incorporating culturally and personally relevant and appropriate strategies into behavior change interventions supports successful outcomes [69], participatory research with teens in rural communities is an ideal approach for developing an understanding of issues that influence behaviors from their perspective, and is a key step in intervention development. Reducing obesity risk and improving the health and well-being of teens in rural communities are important issues, with key implications for public health. Developing obesity prevention interventions tailored to teen perceptions, needs, and interests is key step towards preventing obesity and helping teens living in rural communities develop foundational habits to reduce risk of lifestyle-related chronic diseases, both now and in the future.

Strengths of this study include a large sample size and trained interviewers who were skilled at probing and prompting to elicit deeper insights and understandings regarding this topic. Further, the study sample was diverse; families were recruited from multiple rural counties in Texas, the study included both males and females, and teens who had various levels of physical activity. A focused approach to reducing bias also enhances confidence in the findings. A limitation of the study is that the findings are not generalizable to other teens, although the findings are consistent with those in the literature and provide unique insights that can be further investigated for relevance to teens in other rural communities. An additional limitation is that findings were not examined by birth sex; however, the purpose of this research was not to identify birth sex differences regarding how living in a rural community influenced diet and physical activity choices; rather, it was intended as an initial investigation into teen perceptions related to this issue, regardless of birth sex. Once these perceptions have been clearly articulated, as in this paper, future research can investigate birth sex differences and whether interventions for teens in rural communities should be tailored to birth sex. A final limitation is not investigating how teens defined rural; asking this question could have provided additional insights with which to interpret the results. 

## 5. Conclusions

Qualitative research with teens living in rural Texas communities provided important insights into their perspectives of how living in a rural community influences their diet and physical activity behaviors. These findings go beyond the general understanding of obesity risk factors and shed light on the unique challenges and opportunities encountered by teens in rural communities around diet and physical activity. These insights are invaluable for informing the development of obesity prevention interventions specifically designed for teens in rural communities that reflect the realities encountered in their daily lives. These insights will provide guidance on factors on which to focus an intervention to enhance personal relevance, thus achieving the ultimate goal of increasing the likelihood of sustained behavior change.

## Figures and Tables

**Table 1:** Parent demographic characteristics.

		%	Frequency
Age	30–49	75.0	30
	≥50	25.0	10
Sex	Male	17.5	7
	Female	82.5	33
Race	Black	7.5	3
	White	90.0	36
	Other	2.5	1
Ethnicity	Hispanic	42.5	17
	Not Hispanic	57.5	23
Marital Status	Married	82.5	33
	Single, never married	2.5	1
	Divorced, separated, widowed	15.0	6

**Table 2:** Parent-reported household characteristics.

		%	Frequency
# Adults in Home	1 to 2	75.0	30
	3 to 4	25.0	10
# Children in Home	1 to 2	75.0	30
	3 to 4	25.0	10
Household Education	<College education	25.0	10
	≥College education	75.0	30
Household Income	<USD 21,000	5.0	2
	USD 21,000–USD 41,000	15.0	6
	USD 42,000–USD 61,000	10.0	4
	>USD 61,000	70.0	28
Vehicle Ownership	Yes	100.0	40
	No	0.0	0
Language Spoken at Home	English	97.5	39
	Spanish	2.5	1

## Data Availability

The datasets generated and/or analyzed during the current study are not publicly available due to concerns regarding privacy, but select data are available from the corresponding author upon reasonable request.
